# Knockdown of circ_0003204 alleviates oxidative low-density lipoprotein-induced human umbilical vein endothelial cells injury: Circulating RNAs could explain atherosclerosis disease progression

**DOI:** 10.1515/med-2021-0209

**Published:** 2021-04-07

**Authors:** Qiuxia Su, Xianhua Dong, Chonghui Tang, Xiaojie Wei, Youguo Hao, Jun Wu

**Affiliations:** University Healthcare Branch II, The First Affliated Hospital of Xiamen University, Xiamen, China; Department of Neurosurgery, The First People’s Hospital of Jiangxia District, Xiehe, Wuhan, Hubei, China; Department of Neurosurgery, Cixi Hospital, Wenzhou Medical University, Cixi, Zhejiang, China; Department of Rehabilitation, Shanghai Putuo People’s Hospital, Putuo People’s Hospital Affiliated to Tongji University, Shanghai, China; Department of Neurology, Central Hospital of Xianyang, No. 78, East People Road, Xianyang 712000, Shanxi, China

**Keywords:** AS, ox-LDL, circ_0003204, miR-330-5p, TLR4, NF-κB

## Abstract

Atherosclerosis (AS) is a serious cardiovascular disease. Circular RNAs (circRNAs) play an important role in the progression of many diseases, including AS. However, the role of circ_0003204 in AS is not clear. Oxidized low-density lipoprotein (ox-LDL)-induced human umbilical vein endothelial cells (HUVECs) were used to construct an AS cell model *in vitro*. Cell viability was assessed using cell counting kit 8 (CCK8) assay. Flow cytometry and caspase-3 activity were used to measure cell apoptosis. The contents of inflammatory cytokines were measured using enzyme-linked immunosorbent assay (ELISA). Oxidative stress marker expression and cell injury marker activity were detected by their corresponding Assay Kits. Besides, the expression levels of circ_0003204, miR-330-5p, and toll-like receptor 4 (TLR4) were tested by real-time polymerase chain reaction (qPCR). The interaction between miR-330-5p and circ_0003204 or TLR4 was examined by dual-luciferase reporter assay and RNA pull-down assay. Western blot (WB) analysis was used to determine the levels of TLR4 protein and nuclear factor-kappa B (NF-κB) signaling pathway-related protein. Our data suggested that ox-LDL could suppress viability and promote apoptosis, inflammatory response, and oxidative stress in HUVECs. circ_0003204 was highly expressed in ox-LDL-induced HUVECs, and its silencing could inhibit ox-LDL-induced HUVECs injury. miR-330-5p could be sponged by circ_0003204, and its inhibitor could reverse the inhibition effect of silenced circ_0003204 on ox-LDL-induced HUVECs injury. Further, TLR4 could be targeted by miR-330-5p, and its overexpression could invert the suppression effect of miR-330-5p on ox-LDL-induced HUVECs injury. The activity of the NF-κB signaling pathway was regulated by the circ_0003204/miR-330-5p/TLR4 axis. Our results indicated that circ_0003204 silencing could alleviate ox-LDL-induced HUVECs injury, suggesting that circ_0003204 might be a novel target for AS treatment.

## 1 Introduction

Atherosclerosis (AS) is a harmful condition caused by the accumulation of fat, blood clots, connective tissue, and calcium carbonate in blood vessels [1,2]. As the early stage of AS is not easy to detect, it is usually diagnosed based on clinical examination when the disease has progressed to a certain level [3]. The etiology of AS is still unknown, but apoptosis and injury of endothelial cells caused by various factors are considered to be the main cause of AS [4,5]. The occurrence of chronic inflammation and oxidative stress is an important basis for judging endothelial cell injury [6,7]. Oxidized low-density lipoprotein (ox-LDL) has been proved to be a vital risk factor for AS, and ox-LDL-induced human umbilical vein endothelial cells (HUVECs) apoptosis and injury have been used as a good way to construct an *in vitro* cell model of AS [8,9]. Therefore, elucidating the causes affecting ox-LDL-induced HUVECs apoptosis and injury is of great clinical significance to reveal the pathogenesis of AS.

Circular RNA (circRNA) is non-coding RNA that has been researched in recent years. It has attracted much attention because of its relatively stable closed-loop structure [10,11]. However, there are few studies on circRNAs in the development of AS at present. Through microarray analysis, Li et al. found that there were 943 differentially expressed circRNAs in ox-LDL-treated HUVECs and normal HUVECs, among which circ_0003204 was significantly upregulated, and qPCR detection results showed good consistency with microarray analysis results [12]. However, the role of circ_0003204 in the development of AS has not been studied.

Increasing evidence suggests that circRNA can act as a competitive endogenous RNA (ceRNA) for microRNA (miRNA) to indirectly mediate messenger RNA (mRNA) expression [13,14]. miR-330-5p has lower expression in many diseases and cancers, and its expression is closely related to the progression of the disease [15,16,17]. Liu et al. showed that miR-330-5p overexpression could inhibit oxidative stress and inflammatory response of ox-LDL-induced macrophages, suggesting that miR-330-5p might be related to AS progression [18]. Toll-like receptor 4 (TLR4) has been shown to be highly expressed in the progression of AS [19]. Nuclear factor-kappa B (NF-κB) is an important downstream signaling pathway mediated by TLR4, which is mainly related to cellular inflammation and apoptosis [20,21]. Therefore, TLR4 is an important regulator in AS progression.

Here, our research aims to explore the role of circ_0003204 in AS progression and clarify its potential molecular mechanism through bioinformatics prediction and experimental verification. The research on the function of circ_0003204 can enrich the deficiencies of circRNA research in the progression of AS. In addition, elucidating circ_0003204 molecular mechanism may provide a new theoretical target for the prevention and treatment of AS.

## 2 Materials and methods

### 2.1 Cell culture, treatment, and transfection

HUVECs were obtained from China Center For Type Culture Collection (CCTCC, Wuhan, China) and cultured in Dulbecco’s modified Eagle’s medium (DMEM; Gibco, Grand Island, NY, USA) containing 10% fetal bovine serum (FBS; Gibco) and 1% penicillin/streptomycin (Gibco) at 37℃ with 5% CO_2_ incubator. When the cells reached 90% confluences, they could be transferred into suitable Petri dishes for subsequent experiments. For the screening of the appropriate treatment concentration and treatment time of ox-LDL (Solarbio, Beijing, China), cells were transferred into the 96-well plate. When the cells grew to 50% confluences, HUVECs were treated with the different concentrations of ox-LDL (50, 100, and 200 μg/mL) for 24 h to screen the optimal treatment concentration, and cells were treated with 100 μg/mL ox-LDL for 12, 24, and 48 h to screen the optimal treatment time. Cell transfection could be done when the cells reached 50% confluences, followed by treatment with 100 μg/mL ox-LDL for 24 h. All plasmids and oligonucleotides were purchased from GenePharma (Shanghai, China) and transfected into HUVECs by Lipofectamine 3000 (Invitrogen, Carlsbad, CA, USA). They were listed as follows: circ_0003204 small interfering RNA and overexpression plasmid (si-circ_0003204 and circ_0003204) or their negative controls (si-NC and pcDNA), miR-330-5p mimic and inhibitor (miR-330-5p and in-miR-330-5p) or their negative controls (miR-NC and in-miR-NC), TLR4 overexpression plasmid (TLR4) and its negative control (pcDNA).

### 2.2 Cell viability assay

Cell counting kit-8 (CCK-8) Assay Kit was bought from GlpBio (Montclair, CA, USA). After treating with ox-LDL or transfecting with plasmids and oligonucleotides for 24 h, HUVECs were digested with trypsin (Solarbio) and inoculated into 96-well plates. After 24 h, CCK8 solution was added into cells and further cultured for 4 h. Finally, the absorbance at the wavelength of 450 nm was detected by a microplate reader, and the cell viability was calculated.

### 2.3 Cell apoptosis assay

Annexin V-fluorescein isothiocyanate (FITC) Apoptosis Detection Kit and Caspase-3 Activity Assay Kit (Beyotime, Shanghai, China) were used to determine the apoptosis rate and caspase-3 activity of cells, respectively. Briefly, HUVECs were digested with trypsin and collected the suspension after treatment or transfection. For apoptosis rate detection, HUVECs lysates were stained with Annexin V-FITC and propidium iodide, and then the apoptosis rate of HUVECs was assessed using a flow cytometer. For caspase-3 activity detection, HUVECs lysates were incubated with acetyl-Asp-Glu-Val-Asp p-nitroanilide (Ac-DEVD-pNA) for 2 h, and the absorbance at 405 nm was detected by a microplate reader.

### 2.4 Enzyme-linked immunosorbent assay (ELISA)

HUVECs were inoculated into 6-well plates, treated with ox-LDL or transfected with plasmids or oligonucleotides for 24 h, and cell supernatant was collected. The contents of interleukin-6 (IL-6), interleukin-1β (IL-1β), and tumor necrosis factor (TNF-α) were detected using IL-6, IL-1β, and TNF-α ELISA Kits (MSK, Wuhan, China), respectively.

### 2.5 Measurement of the expression of reactive oxygen species (ROS) and malondialdehyde (MDA) and the activity of lactic dehydrogenase (LDH)

After treating with ox-LDL or transfecting with plasmids or oligonucleotides, the expression of ROS and MDA and the activity of LDH of HUVECs were determined using ROS, MDA, and LDH Assay Kits (Wanleibio, Wuhan, China), respectively.

### 2.6 Real-time polymerase chain reaction (qPCR)

Total RNAs were extracted using TRIzol reagent (Invitrogen) and reverse-transcribed into cDNA using miScript Reverse Transcription Kit (Qiagen, Dusseldorf, Germany). Next, SYBR Green (Takara, Tokyo, Japan) was used to perform qPCR analysis. β-Actin and U6 were used as internal controls. All primers were exhibited as below: circ_0003204: F, 5′-CCCCAAGATGCTGTTGTCCC-3′, R, 5′-TCCGTGGTTCTGACGTCCC-3′; TLR4: F, 5′-AACCACCTCCACGCAGGGCT-3′, R, 5′-TGATGTCTGCCTCGCGCCTG-3′; β-actin: F, 5′-GAGCGCGGCTACAGCTT-3′, R, 5′-TCCTTAATGTCACGCACGATTT-3′; miR-330-5p: F, 5′-GCCTCTCTGGGCCTGTGTC-3′, R, 5′-CAGTGCAGGGTCCGAGGTAT-3′; U6: F, 5′-CTCGCTTCGGCAGCACATATACT-3′, R, 5′-CGCTTCACGAATTTGCGTGT-3′. Relative expression was calculated using the 2^−ΔΔCt^ method.

### 2.7 Dual-luciferase reporter assay

The fragments of circ_0003204 or TLR4 3′UTR containing the putative wild-type (WT) binding sites and mutated (MUT) binding sites for miR-330-5p were cloned into the pGL3 reporter vectors (Promega, Madison, WI, USA), yielding the circ_0003204 WT/MUT or TLR4 3′UTR WT/MUT reporter vector. miR-330-5p mimic or inhibitor was co-transfected with the reporter vectors into HUVECs. The luciferase activity was measured using Dual-Lucy Assay Kit (Solarbio).

### 2.8 RNA pull-down assay

Pierce RNA 3′ End Desthiobiotinylation Kit (Thermo Fisher Scientific, Waltham, MA, USA) was used for this assay. Biotin-labeled miR-NC (Bio-miR-NC) probe, Bio-miR-330-5p probe, or Bio-miR-330-5p mutant (Bio-miR-330-5p MUT; binding sites were mutated to the complementary sequences) probe were transfected into HUVECs for 48 h. Then, HUVECs were lysed and the cell lysates were incubated with the magnetic beads. The expression of circ_0003204 or TLR4 was determined using qPCR.

### 2.9 WB analysis

The protein was lysed using RIPA lysis buffer (Beyotime), separated on a 10% sodium dodecyl sulfate-polyacrylamide gel electrophoresis gel, and transferred to polyvinylidene difluoride membrane (Roche, Basel, Switzerland). The membrane was blocked with 5% nonfat milk and incubated with primary antibodies against TLR4 (1:1,000, Beyotime), p65 (1:500, Beyotime), phosphorylated-p65 (p-p65; 1:1,000, Beyotime), IκBα (1:1,000, Beyotime), p-IκBα (1:750, Beyotime) or β-actin (1:1,000, Beyotime) at 4℃ overnight. After incubating with secondary antibody (1:2,000, Beyotime), the protein bands were visualized using BeyoECL Plus (Beyotime).

### 2.10 Statistical analysis

All data were presented as mean ± standard deviation. The collected data were processed using SPSS19.0 software (SPSS, Inc., Chicago, IL, USA). Statistical analysis was performed using Student’s *t*-test or one-way analysis of variance analysis. *P* < 0.05 was defined as statistically significant.

## 3 Results

### 3.1 ox-LDL induced HUVECs injury and promoted circ_0003204 expression

**Figure 1:**
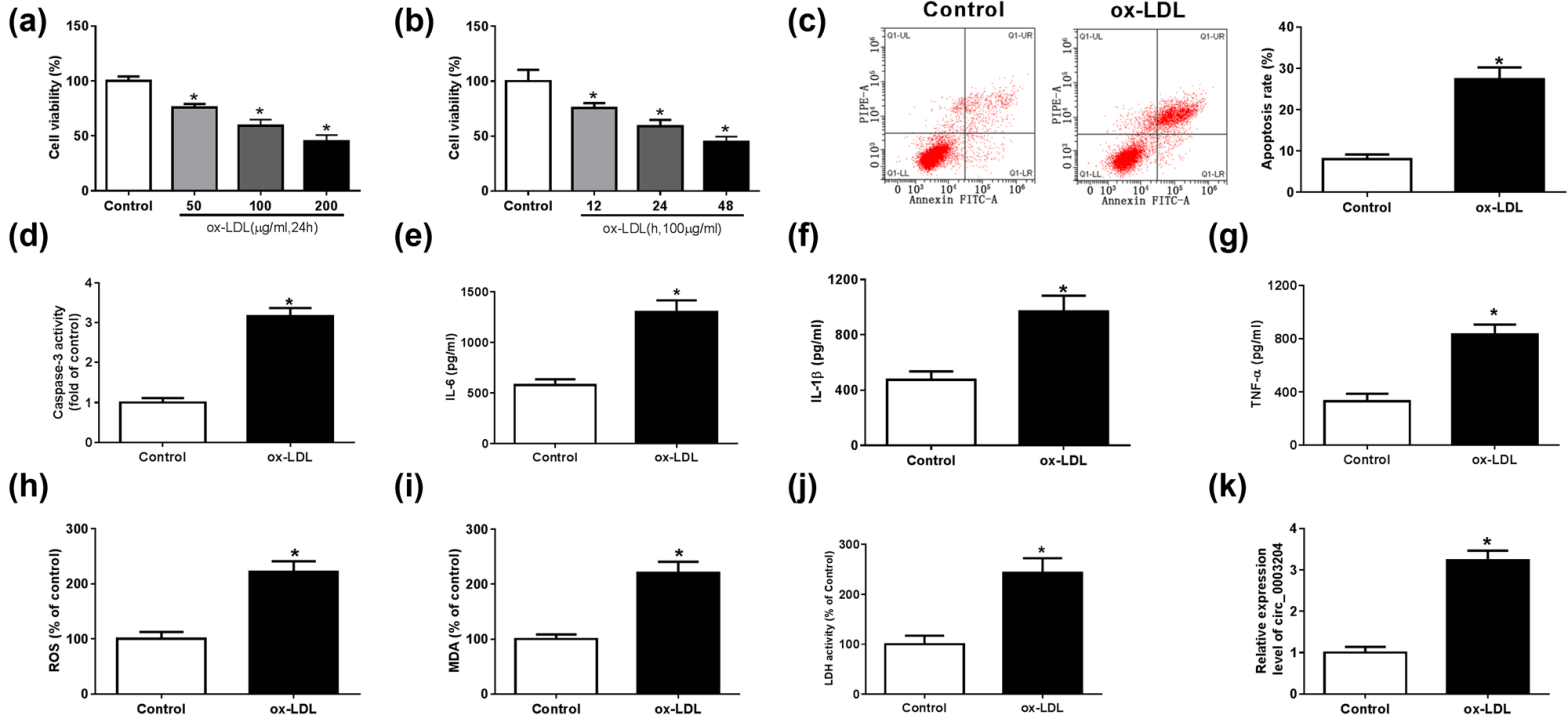
Effects of ox-LDL on HUVECs injury and circ_0003204 expression. (a) HUVECs were treated with 50, 100, and 200 μg/mL ox-LDL for 24 h, and the viability of HUVECs was measured by CCK8 assay. (b) HUVECs were treated with 100 μg/mL ox-LDL for 12, 24, and 48 h, respectively. CCK8 assay was used to detect the viability of HUVECs. (c–k) HUVECs were treated with 100 μg/mL ox-LDL for 24 h. (c) The apoptosis of HUVECs was assessed by flow cytometry. (d) The activity of caspase-3 was determined using Caspase-3 Activity Assay Kit. (e–g) The contents of IL-6, IL-1β, and TNF-α were tested via the IL-6, IL-1β, and TNF-α ELISA Assay Kits, respectively. (h–j) The expression of ROS and MDA and the activity of LDH were measured using the ROS, MDA, and LDH Assay Kits, respectively. (k) The expression of circ_0003204 was determined by qPCR. *P < 0.05.

To determine whether ox-LDL could induce cell injury, we examined the biological function of HUVECs after ox-LDL treatment. By measuring cell viability, we found that ox-LDL could markedly inhibit the viability of HUVECs in a dose-dependent manner and a time-dependent manner (Figure 1a and b). Therefore, 100 μg/mL ox-LDL was used to treat HUVECs for 24 h in further experiments. The results of flow cytometry showed that ox-LDL could remarkably promote the apoptosis of HUVECs (Figure 1c), and the significant increase of caspase-3 activity also confirmed this (Figure 1d). Furthermore, the detection of the IL-6, IL-1β, and TNF-α contents by ELISA assay confirmed that ox-LDL could induce the release of inflammatory cytokines (Figure 1e–g). Moreover, we also found that ox-LDL was able to increase the ROS and MDA expression and the activity of LDH, suggesting that ox-LDL could enhance the oxidative stress and cell injury of HUVECs (Figure 1h–j). Interestingly, we found that circ_0003204 expression was significantly upregulated when ox-LDL-induced HUVECs injury (Figure 1k).

### 3.2 Interference of circ_0003204 alleviated ox-LDL-induced HUVECs injury

**Figure 2:**
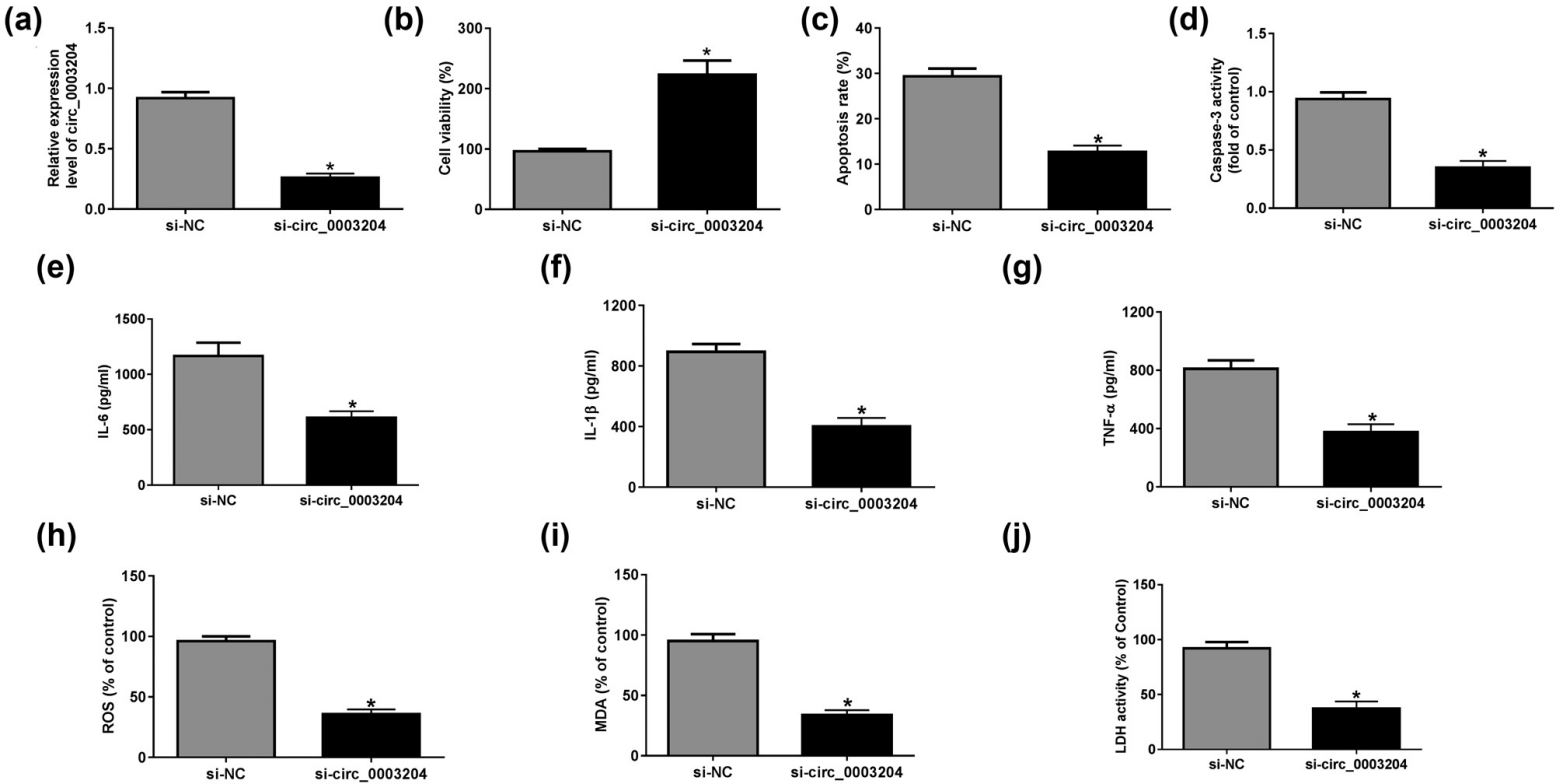
Effects of circ_0003204 silencing on ox-LDL-induced HUVECs injury. HUVECs were transfected with si-circ_0003204 or si-NC, followed by treatment with ox-LDL. (a) The qPCR was used to detect circ_0003204 expression. (b) CCK8 assay was used to determine the viability of HUVECs. (c) Flow cytometry was performed to test the apoptosis of HUVECs. (d) The activity of caspase-3 was measured using Caspase-3 Activity Assay Kit. (e–g) IL-6, IL-1β, and TNF-α ELISA Assay Kits were used to assess the contents of IL-6, IL-1β, and TNF-α in ox-LDL-induced HUVECs. (h–j) The expression of ROS and MDA and the activity of LDH were evaluated using the ROS, MDA, and LDH Assay Kits, respectively. *P < 0.05.

For exploring the function of circ_0003204 in ox-LDL-induced HUVECs, we silenced circ_0003204 expression using si-circ_0003204. The results confirmed that si-circ_0003204 had a good inhibitory effect on circ_0003204 expression (Figure 2a). CCK8 assay results demonstrated that the viability of ox-LDL-induced HUVECs was markedly enhanced by circ_0003204 knockdown (Figure 2b). Besides, silenced circ_0003204 suppressed the apoptosis of ox-LDL-induced HUVECs, as determined by detection of the apoptosis rate and caspase-3 activity of cells (Figure 2c and d). Further, we also uncovered that the silencing of circ_0003204 could inhibit the contents of the inflammatory cytokines IL-6, IL-1β, and TNF-α in ox-LDL-induced HUVECs (Figure 2e–g). By measuring the expression of ROS and MDA and the activity of LDH, we suggested that circ_0003204 knockdown could repress the oxidative stress and cell injury of ox-LDL-induced HUVECs (Figure 2h–j). Therefore, our data indicated that circ_0003204 knockdown might be an effective way to inhibit ox-LDL-induced HUVECs injury.

### 3.3 circ_0003204 served as a sponge of miR-330-5p

**Figure 3:**
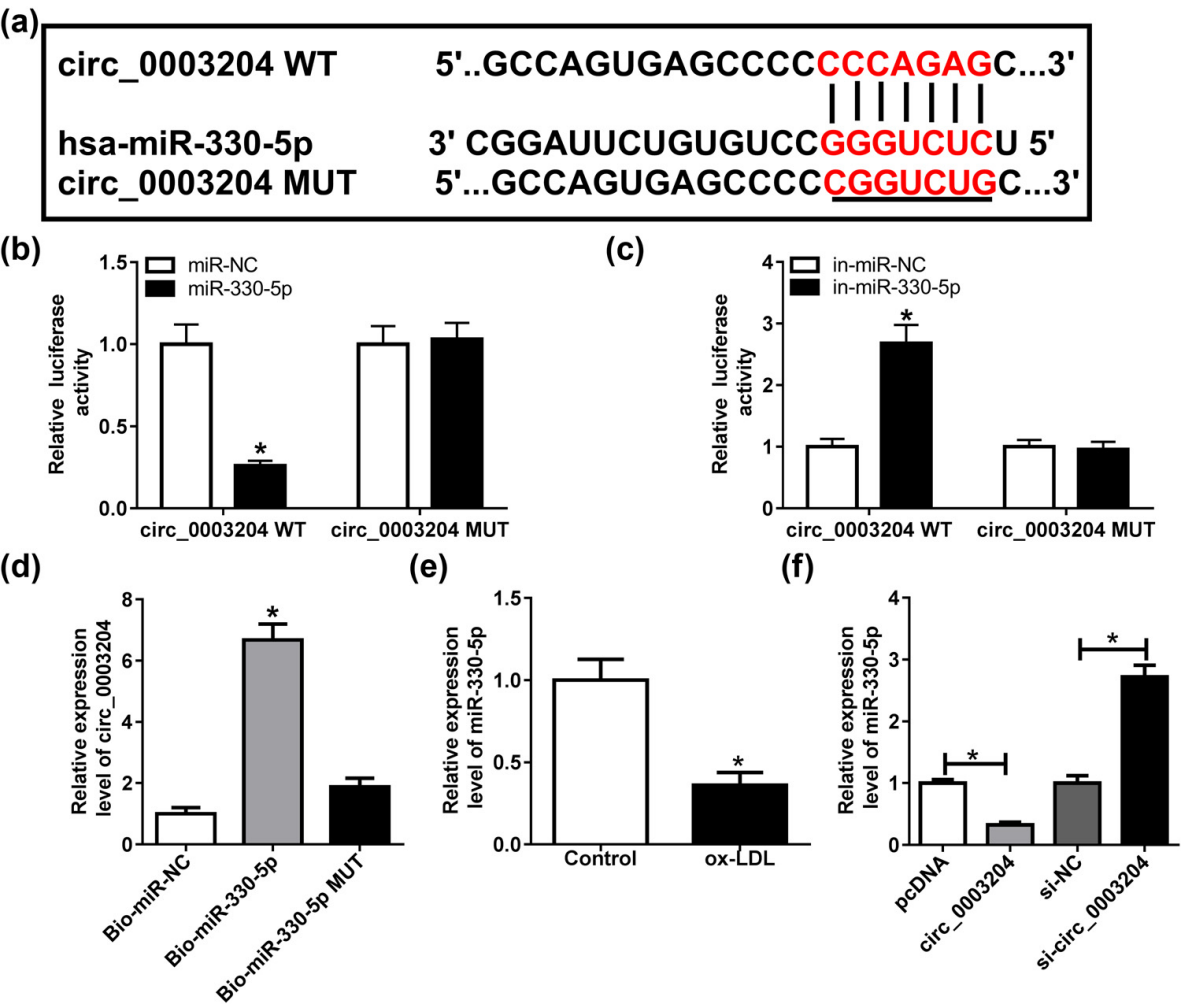
circ_0003204 served as a sponge of miR-330-5p. (a) The fragments of circ_0003204 containing the binding sites or mutant binding sites of miR-330-5p were exhibited. (b and c) Dual-luciferase reporter assay was performed to determine the luciferase activity of circ_0003204 WT or MUT vector in ox-LDL-induced HUVECs. (d) RNA pull-down assay was used to verify the interaction between circ_0003204 and miR-330-5p. The relative expression of circ_0003204 in Bio-miR-330-5p probe or Bio-miR-330-5p MUT probe was detected using qPCR. (e) The miR-330-5p expression in ox-LDL-induced HUVECs or normal HUVECs was assessed by qPCR. (f) HUVECs were transfected with circ_0003204 overexpression plasmid or si-circ_0003204 or their negative controls (pcDNA or si-NC), followed by treatment with ox-LDL. The qPCR was used to test the expression of miR-330-5p. *P < 0.05.

Subsequently, we performed bioinformatics analysis using the Circinteractome tool to predict the targeted miRNAs and found that miR-330-5p contained the binding sites for circ_0003204, as demonstrated in Figure 3a. Then, dual-luciferase reporter assay results showed that miR-330-5p mimic could significantly inhibit the luciferase activity driven by circ_0003204 WT vector, while miR-330-5p inhibitor could markedly promote its luciferase activity, but neither of them affected the luciferase activity driven by circ_0003204 MUT vector (Figure 3b and c). Further, RNA pull-down assay results suggested that circ_0003204 was pulled down by the Bio-miR-330-5p probe but not the Bio-miR-330-5p MUT probe, indicating that there was an interaction between circ_0003204 and miR-330-5p (Figure 3d). Next, we observed that miR-330-5p was remarkably lower expressed in ox-LDL-induced HUVECs (Figure 3e). In addition, miR-330-5p expression was hindered by circ_0003204 overexpression and enhanced by circ_0003204 silencing (Figure 3f). These results implied that miR-330-5p could serve as a target of circ_0003204.

### 3.4 circ_0003204 regulated ox-LDL-induced HUVECs injury by targeting miR-330-5p

**Figure 4:**
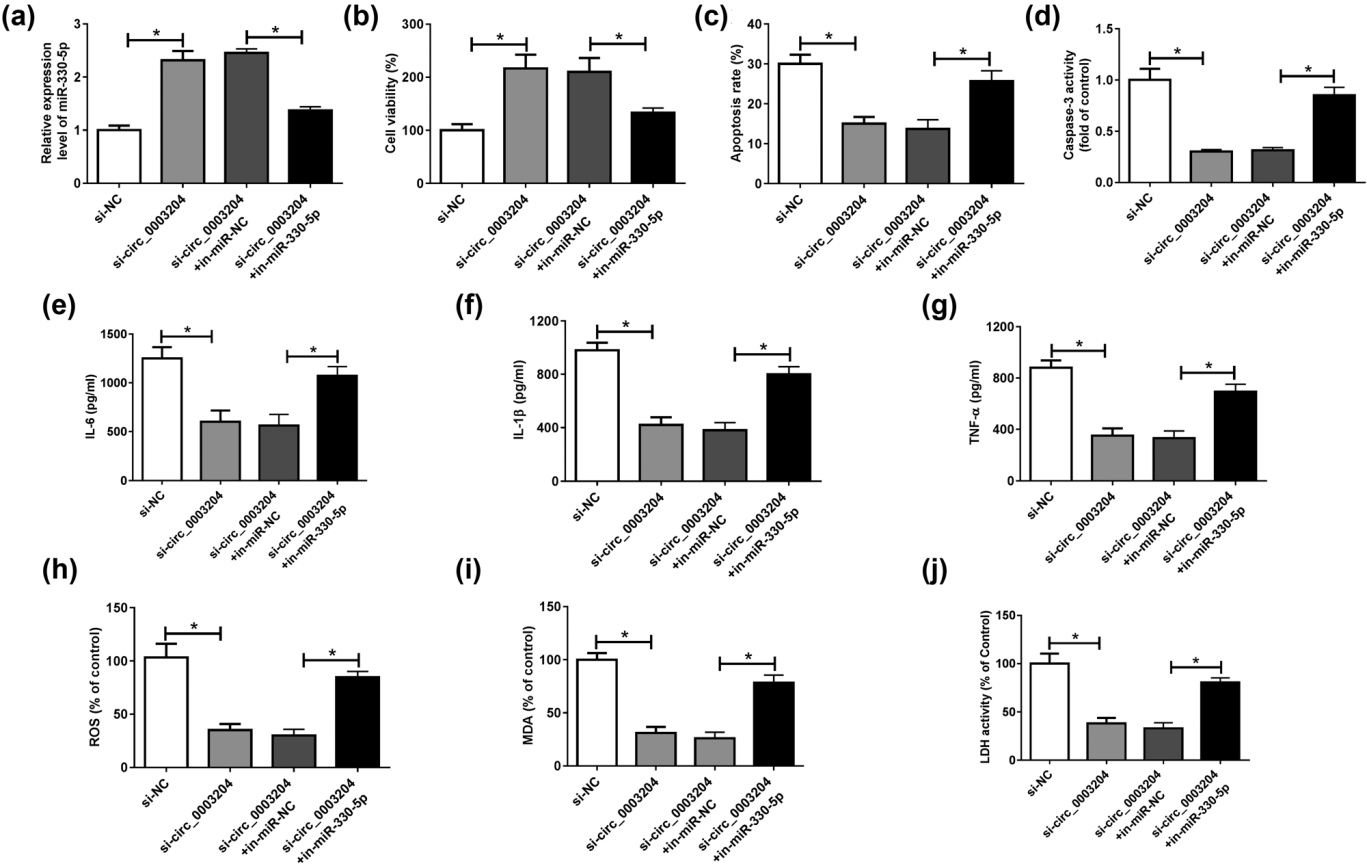
Effects of miR-330-5p inhibitor on ox-LDL-induced HUVECs injury. HUVECs were transfected with si-NC, si-circ_0003204, si-circ_0003204 + in-miR-NC, or si-circ_0003204 + in-miR-330-5p, followed by treatment with ox-LDL. (a) The expression of miR-330-5p was measured using qPCR. (b) CCK8 assay was performed to test the viability of HUVECs. (c) The apoptosis of HUVECs was determined via flow cytometry. (d) Caspase-3 Activity Assay Kit was used to assess the activity of caspase-3. (e–g) The contents of IL-6, IL-1β, and TNF-α were detected using the IL-6, IL-1β, and TNF-α ELISA Assay Kits, respectively. (h–j) The ROS, MDA, and LDH Assay Kits were used to measure the expression of ROS and MDA and the activity of LDH. *P < 0.05.

For exploring whether miR-330-5p was involved in the regulation of circ_0003204 on ox-LDL-induced HUVECs injury, we carried out the rescue experiments using miR-330-5p inhibitor. As shown in Figure 4a, in-miR-330-5p could reverse the promoting effect of si-circ_0003204 on miR-330-5p expression, indicating that its transfection efficiency was better. CCK8 assay suggested that the increasing effect of circ_0003204 silencing on the viability of ox-LDL-induced HUVECs could be inverted by miR-330-5p inhibitor (Figure 4b). Moreover, miR-330-5p inhibitor also could recover the decreasing effect of circ_0003204 knockdown on the apoptosis rate and caspase-3 activity of ox-LDL-induced HUVECs (Figure 4c and d). Besides, the contents of IL-6, IL-1β, and TNF-α suppressed by circ_0003204 silencing could be reversed by miR-330-5p inhibitor, suggesting that miR-330-5p inhibitor increased the inflammatory response of ox-LDL-induced HUVECs (Figure 4e–g). At the same time, the inhibitory effect of silenced circ_0003204 on the expression levels of ROS and MDA, as well as the LDH activity in ox-LDL-induced HUVECs could also be inverted by miR-330-5p inhibitor (Figure 4h–j). Our data revealed that circ_0003204 modulated the viability, apoptosis, inflammatory response, and oxidative stress of ox-LDL-induced HUVECs via sponging miR-330-5p.

### 3.5 TLR4 was a target of miR-330-5p

**Figure 5:**
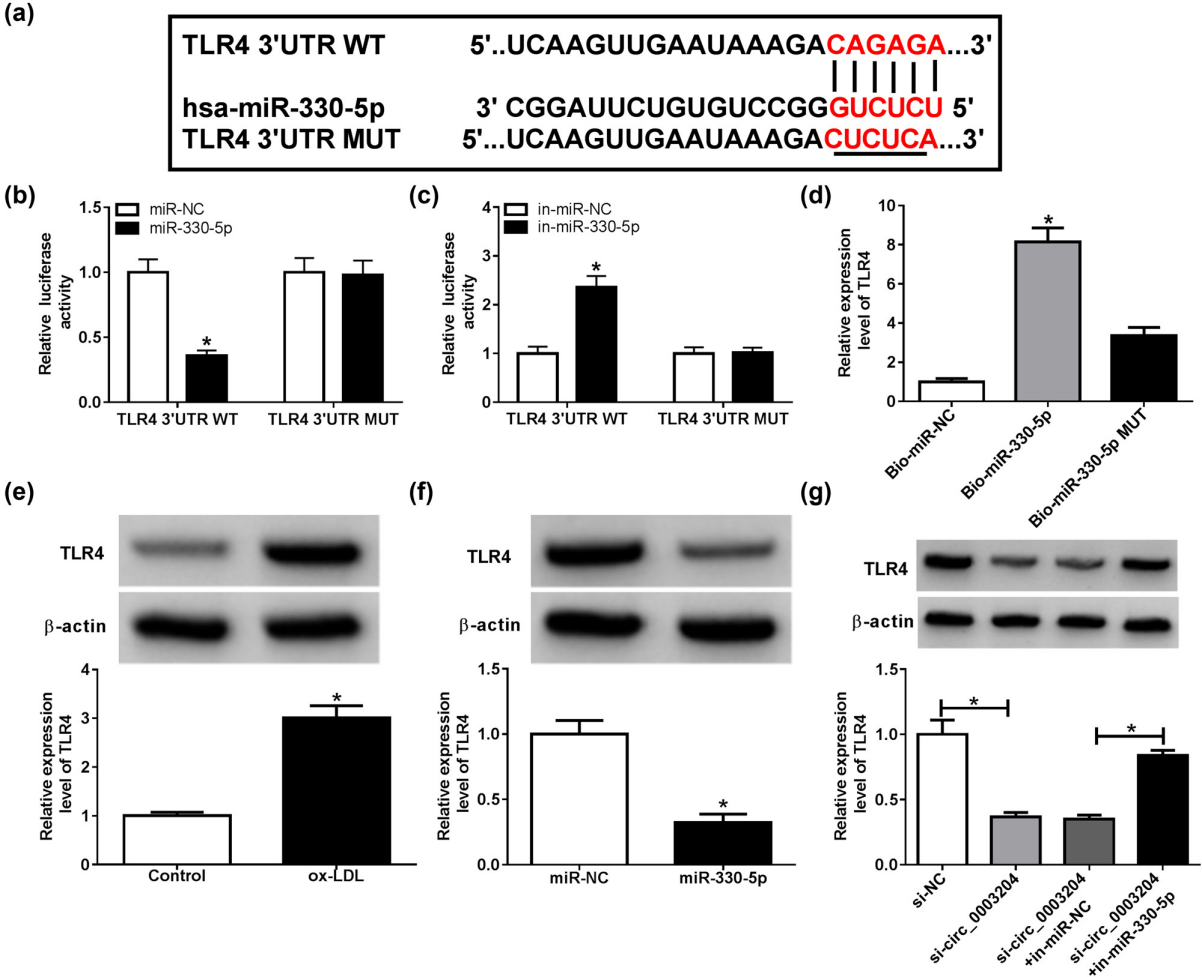
TLR4 was a target of miR-330-5p. (a) The sequences of TLR4 3′UTR contained the binding sites or mutant binding sites of miR-330-5p were presented. (b and c) The luciferase activity of TLR4 3′UTR WT or MUT vector in ox-LDL-induced HUVECs was measured using dual-luciferase reporter assay. (d) The interaction between TLR4 and miR-330-5p was confirmed via RNA pull-down assay, and the relative expression of TLR4 in Bio-miR-330-5p probe or Bio-miR-330-5p MUT probe was determined using qPCR. (e) The protein level of TLR4 in ox-LDL-induced HUVECs or normal HUVECs was detected by WB analysis. (f) The protein level of TLR4 was tested using WB analysis to evaluate the effect of miR-330-5p mimic on TLR4 expression. (g) HUVECs were transfected with si-NC, si-circ_0003204, si-circ_0003204 + in-miR-NC, or si-circ_0003204 + in-miR-330-5p, followed by treatment with ox-LDL. WB analysis was performed to assess the expression of TLR4. *P < 0.05.

Using the DIANA tool, conserved binding sites between miR-330-5p and TLR4 are shown in Figure 5a. The results of dual-luciferase reporter assay indicated that the luciferase activity driven by TLR4 3′UTR WT vector could be restrained by miR-330-5p mimic and enhanced by miR-330-5p inhibitor, while the luciferase activity driven by TLR4 3′UTR MUT vector was not affected by any factor (Figure 5b and c). Further, TLR4 also could be pulled down by the Bio-miR-330-5p probe rather than the Bio-miR-330-5p MUT probe (Figure 5d). Through measuring the expression of TLR4, we discovered that ox-LDL could remarkably promote TLR4 expression in HUVECs (Figure 5e). Furthermore, miR-330-5p overexpression could repress the protein level of TLR4 in ox-LDL-induced HUVECs (Figure 5f). In addition, we found that circ_0003204 silencing could inhibit TLR4 expression, while this effect could be reversed by miR-330-5p inhibitor (Figure 5g). Together, we suggested that miR-330-5p could target TLR4.

### 3.6 TLR4 overexpression reversed the regulation of miR-330-5p mimic on ox-LDL-induced HUVECs injury

**Figure 6:**
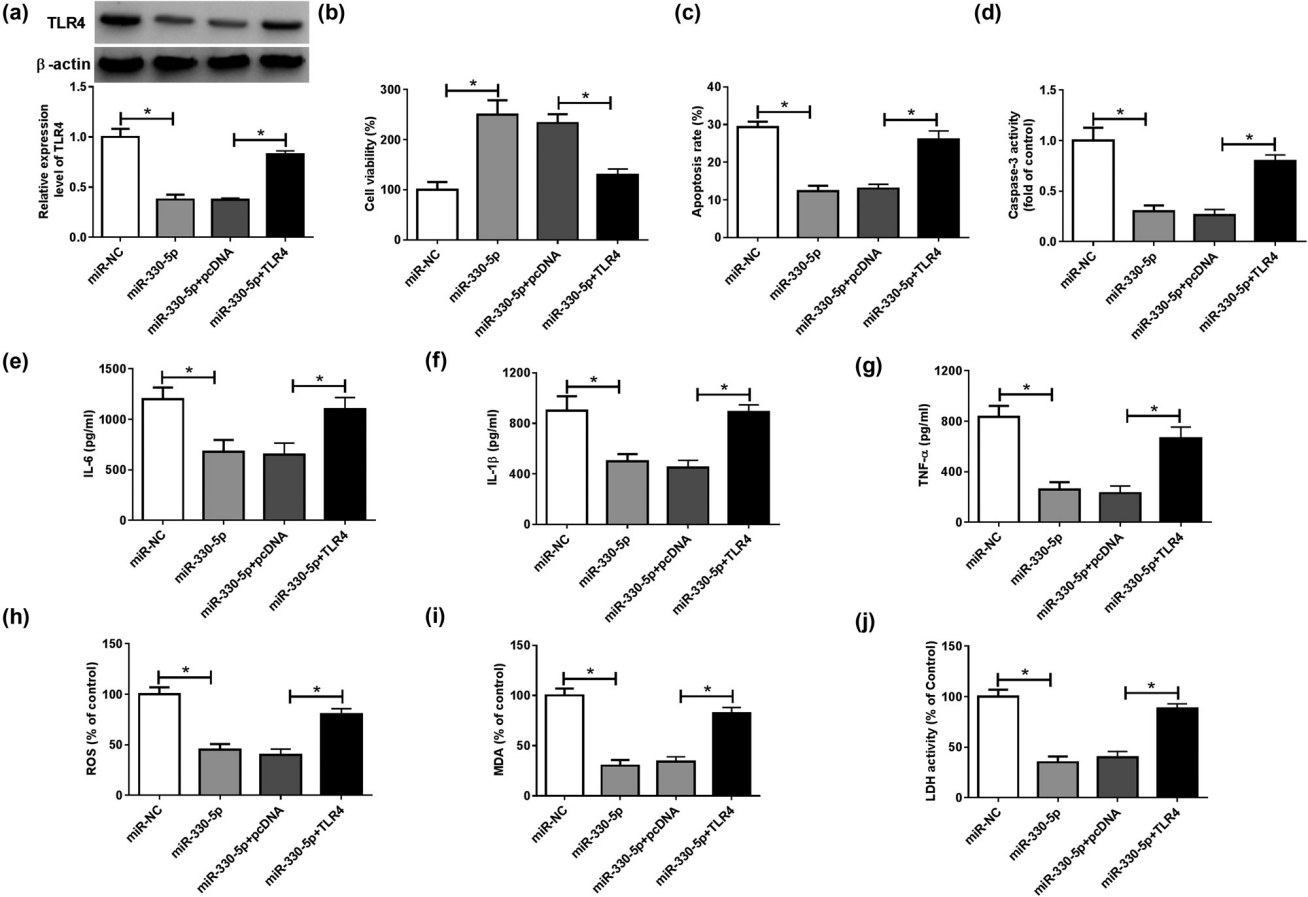
Effects of miR-330-5p mimic and TLR4 overexpression on ox-LDL-induced HUVECs injury. HUVECs were transfected with miR-NC, miR-330-5p, miR-330-5p + pcDNA, or miR-330-5p + TLR4, followed by treatment with ox-LDL. (a) The protein level of TLR4 was determined using WB analysis. (b) The viability of HUVECs was detected via CCK8 assay. (c) The apoptosis of HUVECs was measured using flow cytometry. (d) Caspase-3 Activity Assay Kit was used to determine the activity of caspase-3. (e–g) The IL-6, IL-1β, and TNF-α ELISA Assay Kits were used to detect the contents of IL-6, IL-1β, and TNF-α, respectively. (h–j) The expression of ROS and MDA and the activity of LDH were assessed using the ROS, MDA, and LDH Assay Kits, respectively. *P < 0.05.

To verify that miR-330-5p regulated ox-LDL-induced HUVECs injury was achieved by regulating TLR4, we co-transfected miR-330-5p mimic and TLR4 overexpression plasmid into HUVECs, followed by treatment with ox-LDL. The detection of the TLR4 protein level results showed that TLR4 overexpression plasmid could recover the inhibition effect of miR-330-5p overexpression on TLR4 expression, which revealed that the transfection of miR-330-5p mimic and TLR4 overexpression plasmid was successful (Figure 6a). The results of the CCK8 assay suggested that miR-330-5p overexpression increased the viability of ox-LDL-induced HUVECs, while this effect could be reversed by overexpression of TLR4 (Figure 6b). In addition, overexpressed TLR4 could invert the suppression effect of miR-330-5p overexpression on the apoptosis rate and the caspase-3 activity of ox-LDL-induced HUVECs (Figure 6c and d). Moreover, we found that overexpression of miR-330-5p restrained the contents of inflammatory cytokines, including IL-6, IL-1β, and TNF-α, and TLR4 overexpression also could reverse this effect (Figure 6e–g). In addition, the oxidative stress markers ROS and MDA levels suppressed by miR-330-5p overexpression could be recovered by elevated TLR4 expression in ox-LDL-induced HUVECs (Figure 6h–i), and the inhibition effect of miR-330-5p on the activity of LDH also could be recovered by TLR4 overexpression (Figure 6j). These data suggested that miR-330-5p could regulate ox-LDL-induced HUVECs injury via targeting TLR4.

### 3.7 The circ_0003204/miR-330-5p/TLR4 axis regulated the activity of the NF-κB signaling pathway

**Figure 7:**
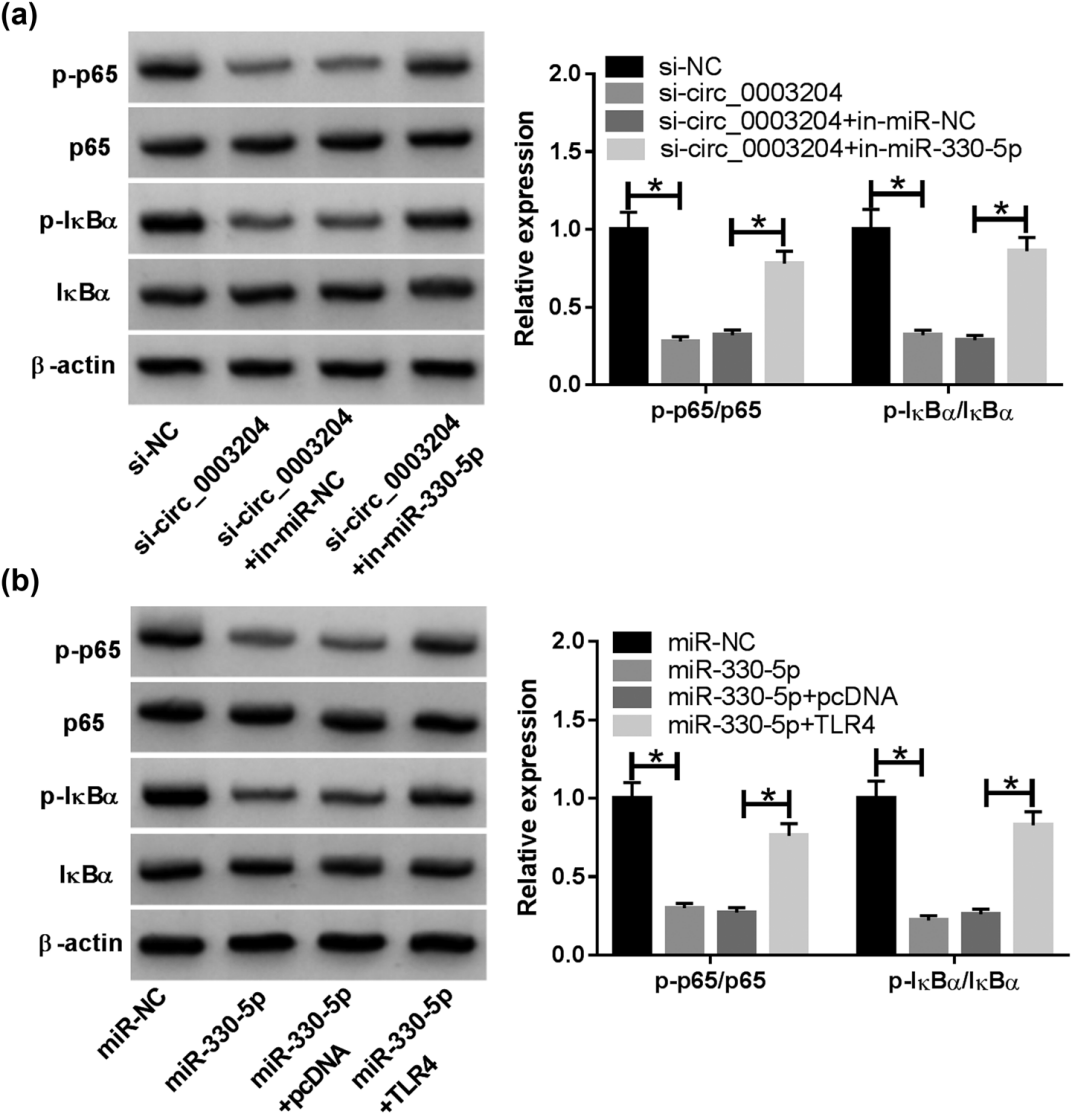
The circ_0003204/miR-330-5p/TLR4 axis regulated the activity of the NF-κB signaling pathway. (a) HUVECs were transfected with si-NC, si-circ_0003204, si-circ_0003204 + in-miR-NC, or si-circ_0003204 + in-miR-330-5p, followed by treatment with ox-LDL. The relative expression of p-p65/p65 and p-IκBα/IκBα was measured using WB analysis. (b) HUVECs were transfected with miR-NC, miR-330-5p, miR-330-5p + pcDNA, or miR-330-5p + TLR4, followed by treatment with ox-LDL. WB analysis was used to determine the relative expression of p-p65/p65 and p-IκBα/IκBα. *P < 0.05.

**Figure 8:**
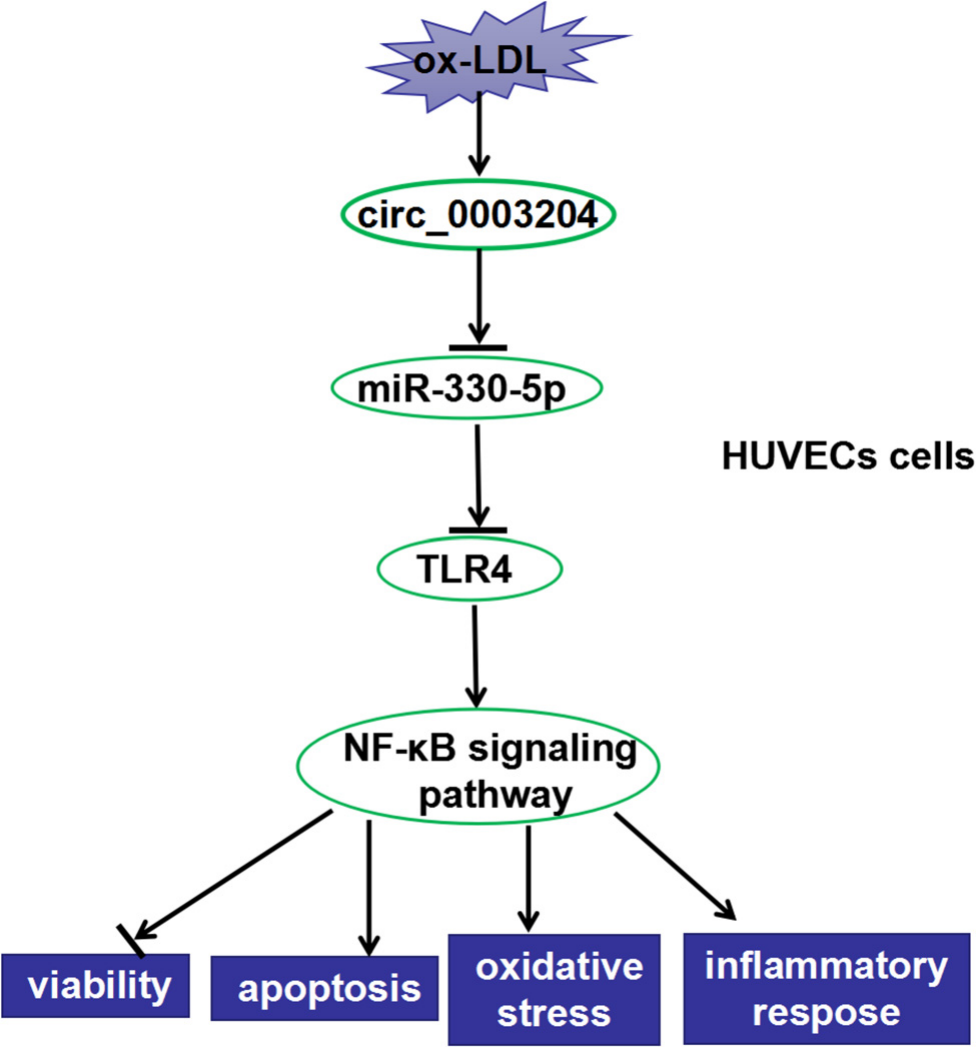
The schematic diagram of the main findings of this research. In ox-LDL-induced HUVECs, circ_0003204 inhibited the viability and promoted the apoptosis, oxidative stress, and inflammatory response of cells via mediating the activity of NF-κB signaling pathway by regulating the miR-330-5p/TLR4 axis.

The phosphorylation of p65 and IκBα is the hallmark event of activation of the NF-κB signaling pathway [22]. To investigate whether the circ_0003204/miR-330-5p/TLR4 axis could modulate the activity of the NF-κB signaling pathway, we detected the relative expression of p-p65/p65 and p-IκBα/IκBα in ox-LDL-induced HUVECs. As shown in Figure 7a, silenced circ_0003204 restrained the relative expression of p-p65/p65 and p-IκBα/IκBα, while this effect could be reversed by miR-330-5p inhibitor. Similarly, the inhibition effect of miR-330-5p mimic on the relative expression of p-p65/p65 and p-IκBα/IκBα also could be inverted by TLR4 overexpression (Figure 7b). These indicated that circ_0003204/miR-330-5p/TLR4 axis regulated the viability, apoptosis, oxidative stress, and inflammatory response of ox-LDL-induced HUVECs by mediating the NF-κB signaling pathway (Figure 8).

## 4 Discussion

At present, preventing the further development of AS from extending the life of patients is the basic policy of AS treatment. Studies have confirmed that anti-inflammatory agents and sonodynamic therapy may be potential treatments for AS [23,24]. With the deepening of research, researchers are also exploring new targets for the treatment of AS. circRNA has received a lot of attention as a potential therapeutic target for many diseases, mainly because its expression is closely related to disease progression [25,26]. For example, circ-YOD1 was considered as a biomarker for coronary artery disease [27], and circDLPAG4 was identified as a potential therapeutic target for myocardial ischemia [28]. In AS, Yang et al. confirmed that circCHFR was abnormally expressed in ox-LDL-modulated vascular smooth muscle cells and was involved in cell proliferation and migration, suggesting that it might be associated with the progression of AS [29]. Li et al. reported that circ_0003575 participated in the proliferation and angiogenesis ability of ox-LDL-induced HUVECs [12].

Our study confirmed the high expression of circ_0003204 in ox-LDL-induced HUVECs, which was agreed with the results of previous studies [12]. Subsequently, circ_0003204 knockdown promoted the viability and inhibited the apoptosis, inflammatory response, and oxidative stress of ox-induced HUVECs, which confirmed that circ_0003204 expression had a positive effect on the progression of AS. More importantly, these results also suggested that the silencing of circ_0003204 might be an effective treatment for AS.

As previously described, circRNA could function as a ceRNA to sponge miRNA [13,14]. Through performing bioinformatics analysis, we found that miR-330-5p had a binding site with circ_0003204. Previous studies have shown that increased miR-330-5p expression could lead to the instability of carotid plaques, indicating that miR-330-5p might be a biomarker for AS treatment [30]. Herein, miR-330-5p inhibitor reversed the suppression effect of circ_0003204 knockdown on ox-LDL-induced HUVECs injury, which further verified that miR-330-5p could be targeted by circ_0003204. Similar to the results of Liu et al. [18], we also found that miR-330-5p overexpression could restrain the HUVECs injury induced by ox-LDL. Our further studies determined that TLR4 was a target of miR-330-5p, which was also confirmed by the reversal effect of TLR4 overexpression on miR-330-5p inhibiting ox-LDL-induced HUVECs injury.

The NF-κB signaling pathway is a classical signaling pathway involved in regulating immune response, inflammatory response, cell differentiation, and apoptosis [22], and its activity is often associated with the expression of TLR4, so it is also called the TLR4/NF-κB signaling pathway [31]. In our study, we demonstrated that circ_0003204 regulated ox-LDL-induced HUVECs injury by regulating the NF-κB signaling pathway activation through the miR-330-5p/TLR4 axis, which was agreed with our previously anticipated results. Of course, we also considered some limitations of this study. In our research, we noticed that the reversal of miR-330-5p inhibitor on the function of circ_0003204 silencing is partial. This indicates that there may be other miRNAs involved in the regulation of circ_0003204 on ox-LDL-induced HUVECs injury, which requires further investigation. In addition, our research has only been verified at the cellular level, and the development of *in vivo* experiments will be our further research direction.

To conclude, our study suggested that circ_0003204 increased TLR4 expression to regulate the viability, apoptosis, inflammation response, and oxidative stress of ox-LDL-induced HUVECs by targeting miR-330-5p.

## Abbreviations

AS: atherosclerosis
ox-LDL: oxidized low-density lipoprotein
HUVECs: human umbilical vein endothelial cells
CCK8: cell counting kit 8
